# Neurophysiological mechanisms of implicit and explicit memory in the process of consciousness

**DOI:** 10.1152/jn.00328.2022

**Published:** 2022-08-31

**Authors:** Don M. Tucker, Phan Luu, Mark Johnson

**Affiliations:** ^1^Department of Psychology, University of Oregon, Eugene, Oregon; ^2^Brain Electrophysiology Laboratory Company, Eugene Oregon; ^3^Department of Philosophy, University of Oregon, Eugene, Oregon

**Keywords:** collothalamic, consciousness, lemnothalamic, memory, phenomenology

## Abstract

Neurophysiological mechanisms are increasingly understood to constitute the foundations of human conscious experience. These include the capacity for ongoing memory, achieved through a hierarchy of reentrant cross-laminar connections across limbic, heteromodal, unimodal, and primary cortices. The neurophysiological mechanisms of consciousness also include the capacity for volitional direction of attention to the ongoing cognitive process, through a reentrant fronto-thalamo-cortical network regulation of the inhibitory thalamic reticular nucleus. More elusive is the way that discrete objects of subjective experience, such as the color of deep blue or the sound of middle C, could be generated by neural mechanisms. Explaining such ineffable qualities of subjective experience is what Chalmers has called “the hard problem of consciousness,” which has divided modern neuroscientists and philosophers alike. We propose that insight into the appearance of the hard problem can be gained through integrating classical phenomenological studies of experience with recent progress in the differential neurophysiology of consolidating explicit versus implicit memory. Although the achievement of consciousness, once it is reflected upon, becomes explicit, the underlying process of generating consciousness, through neurophysiological mechanisms, is largely implicit. Studying the neurophysiological mechanisms of adaptive implicit memory, including brain stem, limbic, and thalamic regulation of neocortical representations, may lead to a more extended phenomenological understanding of both the neurophysiological process and the subjective experience of consciousness.

**NEW & NOTEWORTHY** The process of consciousness, generating the qualia that may appear to be irreducible qualities of experience, can be understood to arise from neurophysiological mechanisms of memory. Implicit memory, organized by the lemnothalamic brain stem projections and dorsal limbic consolidation in REM sleep, supports the unconscious field and the quasi-conscious fringe of current awareness. Explicit memory, organized by the collothalamic midbrain projections and ventral limbic consolidation of NREM sleep, supports the focal objects of consciousness.

## Graphical abstract

**Figure:**
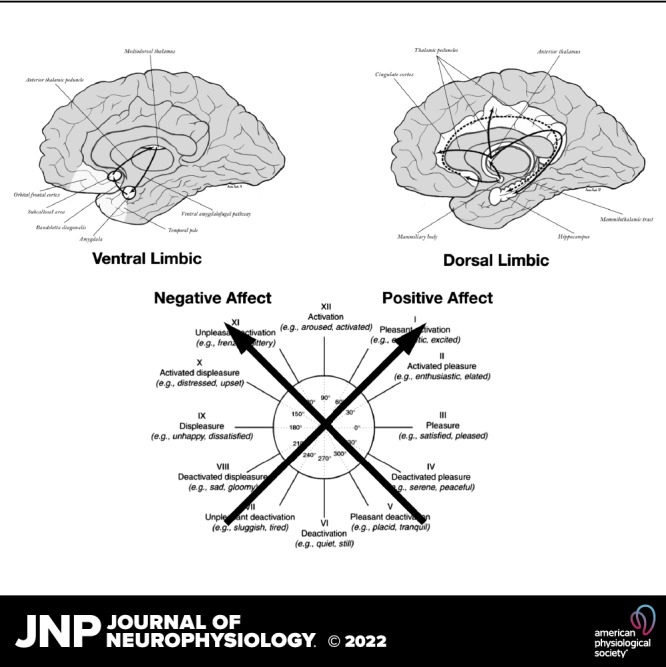

## INTRODUCTION

The structure of the human nervous system is grounded in elementary neural mechanisms for regulating wakefulness and sleep within the brain stem reticular networks that first evolved in early vertebrates (1, 2). Although the regulation of wakefulness may appear to be a trivial aspect of human consciousness, we propose that there is a continuity running from the most basic arousal and sleep controls of the brain stem, to the adaptive consolidation of memory in limbic networks, to the most complex constructions of conscious experience.

Specifically, we propose that regulation by the brain stem reticular formation proceeds through a *lemnothalamic* pathway (3) that engages a vertically integrated regulatory network with a foundation in the brain stem reticular activating system (RAS) with noradrenergic, serotonergic, and cholinergic neuromodulator projections to the forebrain. The control of these projections is organized by lemnothalamic rhomb-di-telencephalic neurophysiological projections, with specific thalamic, hypothalamic, basal forebrain, and dorsal (*archicortical*) limbic and neocortical structures (Luu P, Tucker DM, unpublished observations; Refs. 4, 5). We review evidence that, through this lemnothalamic system, what appear to be elementary controls on sleep and waking exert essential influences on the more complex aspects of implicit memory, including its consolidation in rapid eye movement (REM) sleep. The adaptive operations of implicit memory then shape the affective qualities, and the felt, connotative meaning, of subjective experience.

A parallel and largely separate regulatory mechanism of vertebrate neurophysiology is the *collothalamic* system, based in the collicular networks of the midbrain (including the periaqueductal gray and ventral tegmental area) and propagated by dopamine (DA) projections to the forebrain. These projections target unique structures of the thalamus (mediodorsal), basal forebrain (collopallium), clostro-amygdalar complex, ventral (*paleocortical*) limbic, and ventral neocortical structures (Luu P, Tucker DM, unpublished observations; Refs. 4, 5). This mes-di-telencephalic collothalamic control system regulates not only unique features of affective arousal (anxiety) but also the consolidation of explicit memory in non-REM (NREM) sleep. It is this explicit memory that is most amenable to reflection in ongoing cognition and to communication in language, thereby constituting the most apparent, denotative constructions of consciousness.

This theoretical model, compiled from multiple sources, thus proposes that subjective experience is emergent in each moment from the vertical integration of the complete neuraxis. As a result, the process of consciousness cannot be found solely in those networks of the cerebral cortex that allow higher-order reentrant processing. Rather, the organization of the reentrant conceptual representations that support the emergence of conscious awareness requires the operation of midbrain and brain stem activation and arousal systems that support the adaptive, motivated, organismic memory networks of the corticolimbic hierarchy.

Importantly, these organismic memory networks include both implicit and explicit forms of memory that must be consolidated each night in the unique neurophysiological mechanisms of REM and NREM sleep. As a result, a full, anatomically correct model of the neurophysiology of consciousness implies that the process of subjective experience cannot be understood only through reflecting upon the explicit contents of consciousness. Subjectively, as well as scientifically, we need to understand the implicit fringe of consciousness, continually renewed in the ongoing consolidation of implicit memory, as well as its central focus in present awareness.

Although we arrive at this conclusion through aligning neuropsychological evidence with neurophysiological mechanisms, this realization of the integral role of implicit and contextual aspects of meaning has also been made in classical philosophical and psychological studies of experience, as we review next.

## THE PHENOMENOLOGY OF EXPERIENCE

In the current literature on the neurophysiology of consciousness, researchers have questioned whether animals with varying levels of neural organization could achieve basic elements of consciousness. These elements include attending to the events in the world and monitoring internal states of the organism, whether in avians (6) or agnathans (7). Importantly, depending on your definition of consciousness, such elementary cognitive operations may not satisfy a cardinal requirement of consciousness, which is a second-order neurally *reentrant* monitoring of ongoing experience (8, 9).

Furthermore, even if a neurophysiological account were able to describe the practical tasks of consciousness, together with the reentrant monitoring that constitutes awareness, Chalmers (10) has argued that a mechanistic neurophysiological account would not solve the “hard problem” of consciousness. The hard problem is explaining the subjective qualities or *qualia* of experience, such as seeing a deep blue color, or hearing middle C on a piano. According to Chalmers, even if we had a mechanistic neurophysiological description of the information processing and neural events in vision, or in audition, the subjective qualia would remain unexplained, as if they exist on a different (subjectively distinct) plane from the mechanistic account.

“The hard problem of consciousness is the problem of experience. When we think and perceive, there is a whir of information-processing, but there is also a subjective aspect. As Nagel (1974) has put it, there is *something it is like* to be a conscious organism. This subjective aspect is experience.[…]. Why is it that when our cognitive systems engage in visual and auditory information-processing, we have visual or auditory experience: the quality of deep blue, the sensation of middle C? How can we explain why there is something it is like to entertain a mental image, or to experience an emotion?” [italics in original] (10).

The key term in Chalmer’s argument is *experience*. The problem becomes one of explaining why a certain experience feels the way it does. We propose that the assumption behind the problem, as Chalmers represents it, is that conscious experience and its meaning are fully articulated in the object of conscious experience. There is in fact an a priori dualism in Chalmers’s statement of the problem, which creates something called “experience” that is allegedly a realm unto itself, which can then be peeled off from the brain processes underlying it and admired as a separate irreducible entity.

When experience gets separated from the continuum of organism-environment interactions that generate it, experience then seems to require a different, entirely novel form of explanation, of a sort not possible in the functional accounts of psychology and neurophysiology. Restore a nondualistic, affectively shaped, and value-laden metaphysics of experience, and then consciousness ceases to be something that stands on its own, with its supposedly distinctive mentalistic and thus ineffable properties.

To consider how neurophysiology could possibly be relevant to the hard problem, it may be useful to begin with a philosophical analysis of subjective experience, not as Chalmers’s fixed entity but as a more dynamic mechanism entailing multiple cognitive and affective substrates that the mind must assemble in organizing the process of experience. This more extended approach to understanding consciousness has been taken by the American pragmatist philosophers, including William James and John Dewey.

### John Dewey’s Focus, Field, and Fringe of Consciousness

John Dewey used the term “the Philosophical Fallacy” for the widespread practice of selecting some one or two characteristics of a situation, features that you find it useful to employ given your assumptions and explanatory goals, and then hypostatizing those selected dimensions into alleged ultimate characteristics of every experience. A good example of this fallacy is the aforementioned tendency to assume that the meaning of any particular situation can be made wholly determinate and explicit, thereby ignoring the implicit dimensions of meaning that are integral to the process of subjective experience.

Alfred North Whitehead (11) arrived at a similar conclusion when he reflected on the common error of *misplaced concreteness*, the theoretician’s mistake of making up an abstract construction and then taking it for a concrete, elemental thing in the world.

Crucially, meanings are not just linguistic in nature. Our experience of meaning includes all of the evocations of relevant experience that come to us through gesture, painting, music, architecture, ritual practice, and so on. That’s what makes the arts such marvelous and rich sources of meaning. In each case, meaning includes the significance of the experience for the person.

Moreover, even in linguistic meaning, the meaning of a term, phrase, or clause cannot be captured only by explicit features or relations. Dewey explains, “Apart from language, from imputed and inferred meaning, we continually engage in an immense multitude of immediate organic selections, rejections, welcomings, expulsions, appropriations, withdrawals, shrinkings, expansions, elations and dejections, attacks, wardings off, of the most minute, vibratingly delicate nature. We are not aware of the qualities of many or most of these acts; we do not objectively distinguish and identify them. Yet they exist as feeling qualities, and have an enormous directive effect on our behavior” (Ref. 12, p. 227).

The vast majority of our meaning-making thus goes on beneath conscious attention, but it shapes what does appear in consciousness. Following William James’s description of the *fringe* or *halo* of implicit meaning that surrounds any object or word, Dewey recognized a very useful tripartite structure of meaning that he described as field, fringe, and focus: “If we consider the entire *field* from bright focus through the fore-conscious, the ‘*fringe*,’ to what is dim, sub-conscious ‘feeling,’ the *focus* corresponds to the point of immanent need, of urgency; the ‘fringe’ corresponds to things that just have been reacted to or that will soon require to be looked after, while the remote outlying *field* corresponds to what does not have to be modified, and which may be dependably counted upon in dealing with imminent need” (Ref. 12, p. 235–236; italics added).The focus is what we are currently attending to, marking different qualities, comparing relations, and tracing out consequences. The fringe is the range of highly relevant meanings, relative to the current context, that are readily available possible meanings. The field consists of the further-out horizon of possible meanings that could be appropriate in one’s inquiry but typically are not activated until our inquiry moves to a new focus by following out implications of the focal meaning.

The focus–fringe–field pattern of functional organization of experience applies to perception, conceptualization, and thinking alike. Perceptual events are continuous with conceptual events, so that the transition from perception to conceptual thought is fluid and connected, even though new qualities or properties may arise as conception emerges. This pattern manifests a *principle of continuity* that lies at the heart of naturalistic theories of mind, thought, and language. There are no discrete, independent atoms of perception, conception, or experience. Everything is set within an implicit, generative background of interrelations of objects, qualities, and events. Any given concept or thought involves a field of stable cultural background meanings, a fringe of currently operative meanings of contextual affordances, and an object or event of current focal interest and sustained attention.

Thus in Dewey’s analysis there is a continuity between the field of relatively stable horizonal meanings that operate beneath conscious awareness, the fringe of working meanings of which we are only marginally conscious, and the fully conscious focal object or meaning that is of relevance to our present experience, interests, and values. To celebrate specific qualia of experience is to miss the point of the mind’s phenomenal process.

### William James’s Stream of Consciousness

With a similar consideration for the fringe of awareness, William James used the s*tream of thought* metaphor to capture the fluid, connected, and interpenetrative temporal extent of nonconscious processes that generate what we experience as the ongoing flow of conscious experience (Ref. 13, chapter 9). Similar concepts of the dynamic progression of consciousness in time are found in traditional Buddhist philosophy. These concepts continue to inform the teaching of mindfulness today (14). The account of subjective experience in these traditions recognizes the fluid nature of the mind’s process, with consciousness as an ongoing, evolving development rather than a fixed extraction of an invariant object to exemplify the crystallized, reified, quale.

### Capturing Implicit Meaning in the Communication of Experience

Both James and Dewey looked to science to inform the empirical, pragmatic foundations of philosophy. Extending their contributions to an empirical phenomenology is the work of Eugene Gendlin. As both philosopher and psychotherapist, Gendlin devoted a lifetime of research to the workings of implicit meanings that underlie our conscious thinking. The result was rich examples in how we use language to understand and describe conscious experience on an everyday basis. In the process of language, explicit meanings are emergent from, and continually dependent on, the larger field of implicit meaning.

As psychotherapist, Gendlin understood how meaning involves the personal feelings that give significance to experience. Beginning with the complex domains of meaning signified by our feelings, what can be made explicit in our thinking are the distinctions, concepts, representations, and propositions by which we selectively focus on specific aspects of any situation. But, Gendlin observes, what we can explicitly express always operates in and interacts with the implicit and developing sense of the situation in which you find yourself. “Language brings patterns and distinctions, but what it says exceeds them” (Ref. 15, p. 3). Gendlin indicates the presence of the *more*—the excess of meaning that escapes conceptual capture—by using the word “that” followed by parentheses and four dots, to indicate the more that is working in any growing situation.

Gendlin emphasizes that explicit and implicit meaning work together in many language contexts. Without the implicit that (….), no representational structure or selected pattern would get embedded in and oriented toward the developing meaning, while, without the explicit structural grasping of significant patterns, the implicit that (….) would be no more than a mush of indeterminate felt sense.

There would be selective discrimination but without any motivational directedness and *carrying forward* of the developing meaning of the situation at hand. The metaphors of intertwining, blending, and interanimation all strive to express the essential working together of the explicit and the implicit in the growth of meaning of a situation and the carrying forward of thought in a motivated, grounded, and relevant way.

Gendlin concludes: “We need not wish for convenient givens, and then despair. We can recognize from the start that experience (….) is not given in already-formed units which cognition could simply observe, represent, or approximate. Experience is non-numerical and multischematic[….] Not only does experience (….) not come in cognitive units; we will also recognize that it is always open for further living and action. And, not just open; it often demands further steps[….] Experiencing offers neither the convenience of finished givens, nor the convenience of indeterminacy” (Ref. 15, p. 6–7).

The importance of this nuanced account of the implicit and explicit dimensions of the meaning of a situation is that, to a careful phenomenological analysis, consciousness is not an all or nothing, on or off, thing. The meaning, constantly developing in any particular situation, results from the working together, the enmeshment, the codevelopment, and the mutual engagement of the explicit and the implicit dimensions of meaning. Consciousness thus emerges from a continuum that runs from nonconscious meaning processes, up through marginally conscious feelings (the *felt sense* of a situation), and on up to the full conscious awareness of explicit demarcations and discriminations of meaning.

Consequently, we cannot understand conscious states and processes without appreciating their embeddedness in, and engagement with, implicit, nonconscious meanings that are always in process, so long as we are capable of conscious awareness.

## VERTICAL INTEGRATION OF EXPERIENCE ACROSS THE NEURAXIS

If, then, following James, Dewey, and Gendlin, we understand conscious experience as not only the qualia of pristine, explicit objects of the mind but rather as a dynamic process emerging from a rich array of largely implicit cognition, what neurophysiology could account for this more extended introspection?

Furthermore, if we could study neurophysiology to understand the sources and nature of implicit meaning, could we also gain scientific insight into the process of explicit consciousness that makes subjective experience seem like it exists on a special mental plane, irreducible to biological mechanisms?

We propose there are several levels in the neurophysiology of human self-regulation that influence the process of consciousness, including one (archicortical) memory system specialized for the implicit contextual field of meaning and another (paleocortical) specialized for the articulation of this meaning into the explicit objects of consciousness. We review evidence that the ongoing control of these dual memory systems is grounded in brain stem phasic arousal, for archicortical memory, and midbrain tonic activation, for the paleocortical memory system. These are control systems for regulating waking and sleep that have inherent motive qualities (elation and anxiety, respectively).

### The Higher Reentrant Circuits of the Neocortex and Its Limbic Substrate

Few students of consciousness would object to the importance of brain stem and midbrain mechanisms for the consciousness of being awake or the complementary unconsciousness of being asleep. Nonetheless, the recent neuroscience accounts of neural mechanisms of consciousness often propose that the highest levels of the neural hierarchy are required, involving the reentrant architecture of cortical memory systems (17, 18), the frontothalamic regulation of attention across the cortex (19–21), or the global workspace provided by large-scale neocortical networks (22, 23).

### Yet Consciousness Emerges from the Brain Stem

At the same time, there are compelling theoretical models that have emphasized the subcortical foundations of conscious experience. Merker (24), for example, cited the evidence supporting the *centrencephalic* theory of cerebral function, in which midbrain and brain stem regulation was concluded to be essential to consciousness by Penfield and Jasper after they observed that extensive lesions of various regions of the neocortex did not impair consciousness (at least to the neurosurgeon’s impression) in their epilepsy surgery patients. Merker then provided behavioral observations of hydrocephalic children with very limited cortical development that indicated that at least basic consciousness and social awareness may be mediated almost solely by subcortical systems.

From his extensive study of the neurophysiology and neurochemistry of social behavior in animals, Panksepp (25, 26) assembled a comprehensive neurophysiological theory that situates both human affect and subjective experience within the full architecture of the brain. In this architecture, the basic subcortical control systems of the mammalian brain regulate not only basic emotions but also social attachment, developmental play, and consciousness.

More recently, Solms (27) has organized an array of clinical evidence, including both psychiatric and neurological observations, to attribute the source of consciousness to brain stem and midbrain control systems. Although Solms’s analysis is grounded in the detailed anatomy of subcortical as well as cortical neurophysiology, his clinical observations convey the rich subjective reports of patients with various disorders of experience. This analysis makes it clear that human subjective experience is dependent on a complex hierarchy of regulatory systems of the vertebrate brain, not just one or two uniquely human reentrant networks.

### Overview of the Present Neurophysiological Theory

Our neurophysiological theory follows in this tradition of emphasizing the elementary neurophysiological foundations for even the most complex aspects of human experience. Before reviewing the key evidence on how arousal systems are implicated in the memory consolidation underlying both implicit and explicit forms of consciousness, we first provide an overview of our theoretical argument. Several novel theoretical proposals are introduced in this brief overview; following the overview, a detailed argument citing the supporting evidence on the implicit substrates of the process of consciousness is presented.

#### Reentrance in integrative neocortical networks.

Certainly there are obvious mechanisms of reentrance in the human neocortical architecture that are primary candidates for the generation and maintenance of consciousness. The human frontal lobe, for example, is not only uniquely expanded even among great apes (28) but also uniquely connected to key regulatory circuitry of the thalamus and its reticular nucleus (29). When the frontal pole and its orbital extension are specifically disengaged by absence seizures, the result is a focal and specific loss of conscious intentionality (21, 24, 30). This evidence implies that there may be a uniquely human form of consciousness that requires the uniquely human frontal lobe and its complex representation and control of limbic, thalamic, and brain stem regulatory systems.

Similarly, the posterior human brain includes an interconnected set of convergence zones (31), particularly in midline parietal and posterior cingulate areas (32). These convergence zones form a rich hub of connectivity for integrating multiple perceptual and conceptual representations of working memory into a dynamic, receptive experience of current awareness that is amenable to the reflective reentrance of consciousness (33, 34).

#### Neurophysiology of arousal and sleep.

However, there are also more elementary neurophysiological mechanisms that organize qualitative features of human experience as they regulate basic states of waking and sleep (4, 35). We propose that this regulation of waking consciousness is not a mere biological necessity, but rather it provides integral controls on the structure and process of consciousness that may explain both subtle and explicit qualia of experience. The brain stem and midbrain mechanisms that determine elementary states of sleep and waking turn out to exert qualitative influences on mood and subjective experience as soon as they operate (36, 37).

The lessons of neurophysiology at this level are striking and unintuitive: being awake is not a single process; rather, it combines dual activation and arousal mechanisms with specific affective qualities (38). We have theorized that these affective qualities can be summarized to a first approximation as the *anxiety* that is integral to dopaminergic tonic activation and the *elation* that is integral to noradrenergic phasic arousal (39). As a result, to be awake is to be regulated by some degree of anxiety and some degree of elation, operating in a tensile balance of neurocybernetic process (4).

#### Psychometric evidence of the inherent feelings of activation and arousal.

The behavioral and psychological evidence for this affective quality of neural arousal is strong, reflecting a large literature on the psychometric dimensionality of arousal, mood, and emotion ratings (40). Strong states of activation and arousal in psychopathology have inherent affective qualities (41, 42). The relation to the specific neuromodulators of brain stem and midbrain arousal control systems is also strong, even though the specifics remain controversial. Nonetheless, the evidence from human drug abuse shows that people use stimulants such as amphetamine and cocaine to target the arousal and activation control systems to induce elation artificially (and thereby unavoidably induce anxiety when the elation wanes, thereby propagating the addiction cycle) (39, 43).

#### The structure of experience is controlled differently by tonic activation and phasic arousal.

We review evidence that the operation of the activation and arousal mechanisms leads to regular and predictable changes in the structure of conscious experience. Anxiety focuses consciousness, and elation expands its breadth (39, 44, 45). After reviewing the descriptive evidence, the question becomes: What is the neurophysiology through which these fundamental changes in the scope of consciousness are regulated?

#### Activation and arousal are integral controls on memory consolidation.

We thus emphasize what appear to be specific and differential influences that the brain stem (phasic arousal) and midbrain (tonic activation) systems exert on the limbic control of spatial (contextual) and object (focused) cognition, respectively. The mechanisms of memory may be the engines of specific forms of conscious experience. The lemnothalamic projections from the phasic arousal controls of the brain stem reticular activating system target the dorsal limbic and associated dorsal neocortical networks that provide spatial, contextual cognition. This may explain the broad conceptual scope in the mood/arousal state of elation (Refs. 35, 46; Tucker DM, Luu P, unpublished observations). In contrast, the collothalamic projections from the midbrain preferentially target the ventral limbic and associated ventral neocortical networks that mediate focused attention and the object memory that it supports. This may explain the tight focusing of perception and cognition in states of high anxiety (Refs. 35, 47, 48; Tucker DM, Luu P, unpublished observations).

#### The subcortical and limbic substrates of the process of consciousness.

Remarkably, although the result shapes the structure of conscious experience, the process of these motive controls is largely unconscious. Our moods respond to our perceptions of the events of life, and both our feelings and the structure of consciousness are changed in specific ways. Yet, remarkably, particularly in strong mood states, we do not know this is happening (49).

The process of consciousness that does allow more volitional direction of subjective experience and behavior must engage higher levels of the cerebral networks, particularly those that allow the reentrant processing necessary for ongoing consciousness of the cognitive process (8, 9, 23). The most fundamental of these levels of reentrancy is the architecture of the cerebral hemisphere, with four network levels that are linked in the regular cross-laminar cortical connectivity described by the Structural Model (50). The recent theoretical work formulating the operations of Helmholtz’s unconscious inference within the reentrant corticolimbic architecture (51, 52) provides a powerful insight into how the implicit awareness of the diffuse limbic concepts (at the hemispheric network core) forms an affective envelope for articulating the more discrete and explicit sensorimotor representations at the neocortical level (5).

Because the corticolimbic network dynamics differ for the object memory of the ventral division and the contextual memory of the dorsal division of the mammalian cerebral hemisphere (4), we can understand the specific alignment of the primitive activation (anxiety-focus) and arousal (elation-expansiveness) controls on the structure of consciousness by understanding the differential neurophysiology of the dorsal and ventral divisions of the cerebral hemispheres. Why reentrance yields palpable components of conscious experience at this level is because the corticolimbic networks provide *concepts*: representational capacities at each network level that support the reentrant processing that generates awareness of both the implicit contexts and the explicit objects of consciousness (5, 49).

#### The structure and process of consciousness emerge from the mechanisms of sleep.

To understand the subcortical foundations of these higher reentrant corticolimbic mechanisms for consolidating experience, we theorize that a novel perspective on the differential neurophysiology of the lemnothalamic (dorsal limbic, context) and collothalamic (ventral limbic, object) control systems is provided by the new realization of their specific and differential forms of consolidating of memory in rapid eye movement (REM) and non-REM (NREM) sleep, respectively (Tucker DM, Luu P, unpublished observations). The recent evidence shows that REM sleep stages consolidate implicit memory, consistent with the implicit nature of contextual cognition mediated by the dorsal limbic-neocortical networks. In contrast, NREM sleep stages consolidate explicit memory, consistent with the explicit focus of the object memory of the ventral corticolimbic networks (53–55).

The unique lemnothalamic and collothalamic strategies for developing neural architectures thus appear to be more or less balanced in waking consciousness, with collothalamic differentiation of objects and lemnothalamic integration of contexts. Yet the newly understood functional neurophysiology of sleep shows that these regulatory systems are incompatible in their basic neurophysiological regulation of cortical representations, such that they must alternate to operate separately (yet interactively) in the neurophysiological stages of each night’s sleep.

There are then five or so NREM-REM cycles each night. In each cycle, the neurophysiology of NREM appears to consolidate the explicit memory of recent experience in each cycle, before the neurophysiology of REM organizes (through downscaling the cortical synaptic architecture) the implications of the day’s experience for the implicit memory and emotional integrity of the self (Refs. 54, 56, 57; Tucker DM, Luu P, unpublished observations). This progression in the neurophysiology of sleep suggests it is the lemnothalamic organization of adaptive impulses and contextual memory that is the most fundamental process for preparing the implicit field of the unconscious mind. This implicit, largely lemnothalamic field is then the ongoing adaptive organismic framework not only for spatial contextual memory but for the unfolding process of the more explicit, collothalamic specification of object awareness that is articulated in waking consciousness.

#### Ineffable qualia may be illusions of explicit consciousness.

Thus, as the lemnothalamic system supports the ongoing consolidation of implicit memory, the collothalamic articulation of explicit memory must be the critical process for differentiating the sustained awareness of the explicit discrete objects of consciousness. These objects of consciousness, such as middle C or deep blue, appear to be, for Chalmers and those who have accepted his argument, the ineffable qualia. The neurophysiology of the ventral limbic networks, linked to the claustro-amygdalar motive control of the reentrant basal ganglia regulation of the thalamus, midbrain, and cortex (Luu P, Tucker DM, unpublished observations), provides mechanisms for articulating the delineation of specific objects of experience, to be organized for human cognition in the ongoing verbal stream, the sequenced and ordered cognitive representations of linguistic expression.

The reentrant collothalamic loops coursing through the basal ganglia, pallidum, midbrain, thalamus, and cortex must articulate explicit thoughts not unlike these circuits select and order explicit motor sequences (58, 59) as well as explicit language sequences (60, 61).

#### Toward a neurophysiological phenomenology.

Thus the process of consciousness can be studied in relation to the neurophysiological mechanisms that support the foundations of arousal, cognition, and memory consolidation. Through regulating neural activity, these mechanisms must support the adaptive control of cognition and memory required not just for *homeostasis*, organizing behavior to maintain bodily integrity, but *allostasis*, the anticipation of adaptive needs and challenges (4, 49, 62). In both cases, the *adaptive* operations of neural activation and arousal systems must extend across the vertically integrated neuraxis, bringing visceral requirements, as represented in hypothalamic and limbic regions, to shape the ongoing consolidation of memory in waking and in sleep.

These adaptive controls on activation and arousal are thus integral to the unconscious field of cognition that forms the wider basis for the ongoing process of consciousness. They are the underpinnings of those unique networks of the human brain that allow the extensive frontal pole and the integrative hub of the posterior cingulate and parietal lobes to regulate both corticolimbic and corticostriatal dynamics through thalamic mediation in order to articulate the explicit objects of consciousness. Our goal in the remainder of this review is to bring the neurophysiology of activation and arousal to clarify the differences between explicit and implicit cognition that may encourage a deeper scientific introspection into the process of conscious experience.

## THE NEUROPHYSIOLOGY OF AROUSAL, MOOD, AND MOTIVE CONTROL

Beginning with Bremer’s classic transections of the brain stem (63), it became clear not only that the neurophysiology of arousal is regulated by the most conserved of neural systems in the vertebrate brain stem but also that the control of waking is integrally related to the highly organized neurophysiology of sleep. The characterization of the brain stem reticular activating system (RAS) (2) provided insight into cerebral arousal as a dynamic process, a process that we normally self-regulate to maintain optimal psychological function (64). For neuropsychological theory, arousal as an intrinsic neural self-control mechanism became a much more instructive theory than the popular James–Lange account of psychological arousal as autonomic activity interpreted by a separate (psychological) cognitive process (65).

Nonetheless, the theoretical progress in bringing neurophysiology to psychological theory in the later twentieth century was hampered by the behaviorist reductionism that was inherited by the emerging discipline of neuroscience. As Hobson and McCarley (1) delineated the neuromodulator mechanisms of sleep regulation within the reticular activating system, with the increased activity of cholinergic modulation and abrupt decrease of noradrenergic and serotonergic modulation in the onset of REM sleep, their interpretation not only emphasized the specificity of mechanisms (vs. the more generic RAS concept) but also concluded that, because the neuromodulator mechanisms could be specified, REM dreams are therefore epiphenomena of neurophysiology, not an integral mechanism for organizing human experience.

Importantly, the theoretical progress in neurophysiology in recent decades is clearly overcoming this narrow reductionism, with the neurophysiology of REM providing potential insight into the ways that memory consolidation, under brain stem arousal controls, may be integral to the preparation for active inference and predictive coding in waking cognition (66, 67).

Well before such recent insights, an important contribution of the neurophysiology of arousal to psychological theory came from Pribram and McGuinness’s analysis of neural systems that support *tonic activation*, such as reflected in the readiness potential in motor control, versus *phasic arousal*, such as the brief period of enhanced neural activity in the orienting response to novel sensory stimuli (68, 69). Building on this framework, Tucker and Williamson (39) reviewed the evidence on specific neuromodulator influences on behavior to formulate the unique cognitive controls emergent from these neurophysiological mechanisms of tonic activation and phasic arousal. Supported by the unique effects of dopamine modulation, tonic activation appears to provide a sensitization or *redundancy bias* on ongoing working memory of the forebrain, such that attention becomes focused and sustained to support deliberate and analytic thinking. Importantly, there is an affective state associated with this redundancy and focus: anxiety. The neuropsychological self-regulation achieved by this tonic activation mechanism can explain not only the focusing of attention under conditions of threat but the exaggerated focus, redundancy, and stereotypy of thought and action in the chronic anxiety of obsessive-compulsive disorders (4, 39, 70, 71). More recent theoretical work has aligned this tonic activation mechanism with the collothalamic regulatory system of the ventral limbic division, as outlined below.

The implication for the neurophysiology of consciousness is clear: one major component of being awake (conscious) is associated with both the affective quality of anxiety and the structural organization of awareness to become more focused and differentiated. Classical reports of the pathology of perception in extreme anxiety show focus and discretization of vision to the extreme. A schizophrenic patient in a highly anxious psychotic episode described seeing a corner of a door, or the knob, or the hinge, but not being able to see the whole door (72). The elementary neural control of tonic activation (anxiety) thus alters the very structure of conscious awareness, constraining it to a tight focus.

Contrasting with the redundancy bias of tonic activation is the habituation bias that appears to reflect neuromodulation by the noradrenergic phasic arousal system (39). Here as well, a specific component of the neurophysiology of arousal has a unique influence on the cybernetics of neural activity. Given the inherent spatiotemporal limits of working memory, consciousness may be supported by a broad access to associative memory, which is then inherently short lived (and thus implicit), or it may be supported by a highly focused working memory (and thus sustained and explicit). With its rapid habituation, phasic arousal supports the broad attention of an orienting response. The affective dimension associated with phasic arousal appears to be the *depression-elation* dimension, such that the strong engagement of expansive attention explains the symptomatology of exaggerated elation in clinical mania (35, 39). Here the consciousness of the manic person is modulated by elation to become expansive and holistic, an opposite effect from anxiety in regulating the structure of consciousness.

The implication of this early theoretical work on the neurophysiology of activation and arousal is that the control of waking is not undifferentiated but has specific psychological qualities of motive control (anxiety vs. elation), as well as specific influences on attention and working memory (redundancy and focus vs. habituation and expansiveness) (4, 71). To this way of reasoning, the most elementary neurophysiology of waking consciousness applies inherent controls on both the affective quality of experience and the associated cognitive capacity (4). As reviewed in *The Dimensional Structure of Mood and Arousal*, the affective qualities of the specific tonic activation phasic arousal states are now well documented in the psychological literature.

### The Dimensional Structure of Mood and Arousal

In the important function of elementary consciousness in monitoring the organism’s bodily status (through our feelings), the evidence on the psychological experience of emotion and mood states suggests an integral and predictable relation to the neurophysiology of arousal. The neural control of activation and arousal (being awake, drowsy, or alert) is an affectively charged experience (anxious and/or elated in some measure). The psychometric evidence for this is well established, but it is best understood through a neurophysiological interpretation.

**Figure 1.**
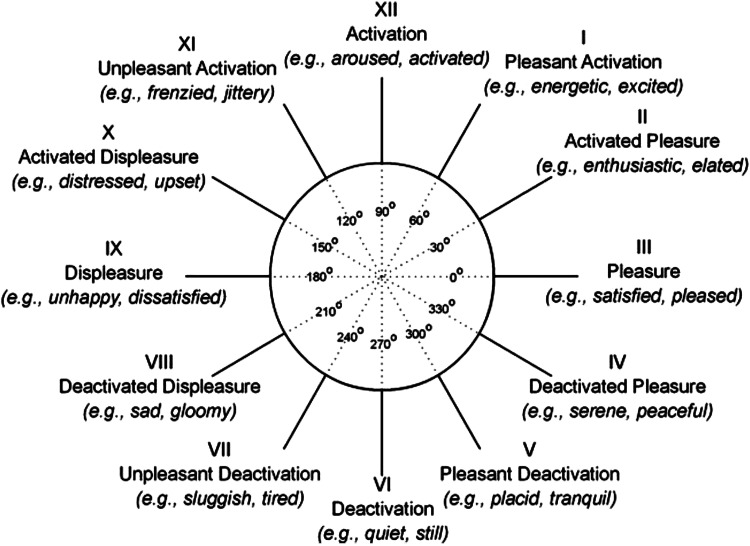
Circumplex of mood and emotional states. From Yik et al. (73). Reproduced with permission.

Classical psychometrics (with factor-analytic dimension reduction) has analyzed the dimensions of emotions in various ratings of mood, emotional states, or personality characteristics to describe a metric space framed by an *arousal* dimension (calm-excited) and a *valence* dimension (good-bad) (73). However, examination of variability on these dimensions, such as during daily experience or in the exaggerated emotions of psychopathology, shows that the actual dimensions describing this space are rotated 45°, describing a positive affect (PA) (elation) dimension (pointing *top right* in Fig. 1) (73) and a negative affect (NA) (anxiety and hostility) dimension (pointing *top left*) (74). Given the nature of factor analysis, both these rotations describe the static correlations equally well, but only the second (PA/NA) rotation fits the evidence on mood and affect changes over time. Thus in Fig. 1 high PA is pleasurable arousal or elation (*top right*) and low PA is unpleasant low arousal or depression (*bottom left*); high NA is unpleasant activation or anxiety (*top left*), and low NA is serenity and safety (*bottom right*).

Tellegen’s (74) analysis of variability was a key insight that motivated this correct rotation: changes in mood, such as variations during the day, do not move along an undifferentiated “arousal” dimension or along an equally abstract “valence” dimension; rather, mood varies from depression to elation (positive affect or PA) and also from security to anxiety/hostility (negative affect or NA). Once we interpreted this well-replicated research finding correctly, we discovered that arousal is inseparable from affect.

Consistent results are obtained in psychometric ratings of normal arousal states during performance of laboratory tasks (75). The experience of arousal varies on two dimensions, one describing being *energetic* (elation) and another describing being *tensed-up*, *jittery* (anxiety) (75).

The implications of this dimensionality of arousal, mood, and emotion are fundamental to both psychology and neurophysiology. Because of the intrinsic neuromodulator control of mood and arousal (as described below), there is no “arousal” separate from influences on mood. There is no positive-negative “valence” of mood state separate from the neurophysiological controls on arousal.

Furthermore, clinical observations of mood variability in personality disorders (76) support the dimensions of PA (varying in clinical mood disorders from depression to elation) and NA (varying from the sense of security to the experience of high anxiety). The cognitive characteristics of strong mood states in descriptive psychopathology (76) are consistent with the theoretical proposal that tonic activation and phasic arousal cause structural changes in consciousness. Not only does elation lead to an expansive and holistic scope of attention in mania, but a similar effect is seen in normal positive affect. Isen (46) and associates have shown that enhanced creativity may be induced by giving a person a small gift. Similarly, when manic individuals were given lithium to normalize their mood, their high scores on a creativity (remote associates) test were lowered to the normal range (77).

In a similar but opposite way, when anxiety is induced experimentally, such as by telling the participant they are in the stress condition of the experiment in which they must listen to white noise, the effect is a loss of holistic scope and a more analytic, detailed approach to perception, classically described as *tunnel vision* (48, 78, 79).

Thus, both psychometric and psychopathological evidence support the theory that elation and anxiety (PA and NA) are the fundamental dimensions of arousal, mood, and the structure of consciousness. Elation expands the scope of working memory and thus conscious experience, whereas anxiety invariably focuses attention and thereby restricts the scope of awareness.

## DUAL LIMBIC DIVISIONS REGULATING EMOTION AND MEMORY

How do these structural changes in consciousness arise with changes in elementary neurophysiological mechanisms of tonic activation and phasic arousal? One important realization from extensive clinical observations is that the mechanism itself is fully unconscious. The depressed person, for example, sees himself and the world as terrible, and may suicide, with no insight into the fact that the mood is causing his dejection and negative appraisal, even when reminded that he was elated just a week earlier (35, 76).

Although we have emphasized that the neuromodulators of the reticular brain stem and midbrain are responsible for these mood dynamics (4, 80), the effects of the motive controls on emotional arousal are organized at each level of the neuraxis, engaging basal forebrain and limbic circuits differentially (Luu P, Tucker DM, unpublished observations; Ref. 4). Particularly important is the functional differentiation between the dorsal, archicortical limbic division (with its associated dorsal neocortex supporting spatial, contextual memory) and the ventral, paleocortical limbic division (with its ventral division of neocortex supporting object memory and focused attention). Because the limbic networks regulate both emotional processes and memory consolidation, they are in a key position for mediating the structural changes in the scope of awareness associated with the neurophysiological arousal controls of elation and anxiety.

Moreover, our recent theoretical work suggests that there is a continuity running from the brain stem and midbrain arousal controls through diencephalic and basal forebrain structures (Luu P, Tucker DM, unpublished observations). The effect is to differentially engage the dorsal limbic function of spatial memory as a function of the current level of elation and to engage the ventral limbic function of object memory as a function of the current level of anxiety (4, 35). By outlining this theoretical model in *Lemnothalamic Regulation of Phasic Arousal, Positive Affect, and Dorsal Limbic Control* and *Collothalamic Regulation of Tonic Activation, Negative Affect, and Ventral Limbic Control*, we set the stage for proposing that there are dual limbic mechanisms for tuning access to the unconscious field of contextual meaning that underlies the more limited focus on the object of consciousness.

### Lemnothalamic Regulation of Phasic Arousal, Positive Affect, and Dorsal Limbic Control

**Figure 2.**
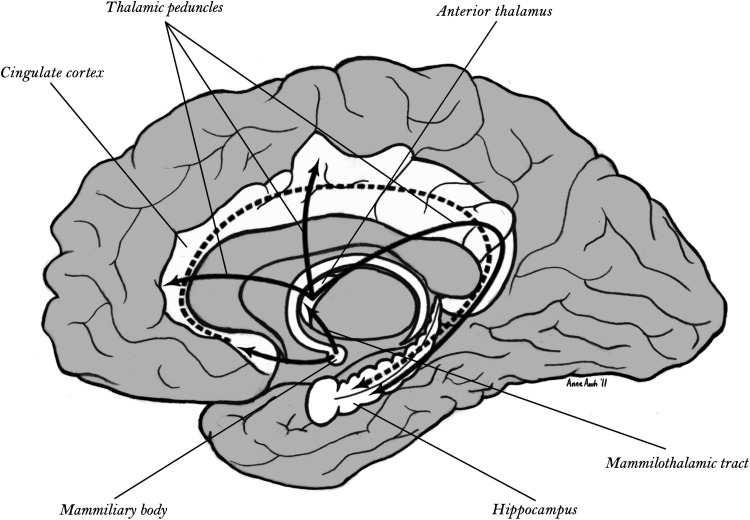
Papez circuit integrating the archicortical limbic division based on the hippocampus, cingulate cortex, and anterior nuclei of the thalamus. From Tucker and Luu (4).

The anatomy of the networks of the human cortex follows the general architecture shown in primate cortex (81, 82). For the dorsal division, the cingulate cortex provides the limbic base, which is then connected to dorsal heteromodal areas (including parietal and frontal), which are connected to unimodal association and then primary sensory and motor areas. From studies of memory in several species including humans, the dorsal division of the cortex appears essential to spatial memory and to the cognitive representation of the spatial context of behavior (83–85). The subcortical limbic base of the cingulate cortex includes the classical Papez circuit, as shown in Fig. 2.

This archicortical network interfaces between the subcortical lemnothalamic control, grounded in the brain stem reticular activating system (RAS), and the structured reentrant architecture of the dorsal division of the cerebral hemisphere. This neocortical architecture, formalized by the Structural Model (50), could be described as *asymmetrically reentrant*, in that projections from higher regions to lower ones (from limbic to heteromodal, from heteromodal to secondary association areas, from secondary association areas to primary sensory-motor cortex) proceed from infragranular layers (5–6) of the cortex to supragranular layers (2–3). In contrast, the reciprocal connections in the opposite flow of corticolimbic traffic arise from supragranular layers (2–3) and target the granular layer (4) (86). The corticolimbic consolidation of memory appears to operate through the communication between cortical representations and the dynamic exercise of those representations by limbic networks including the hippocampus (87), with working memory mediating between limbic representation of interoceptive processing (visceral needs and desires) and the neocortical representation of exteroceptive processing (sensory and motor constraints of the environment) (5, 88, 89). As consciousness gains its substance from working memory, the dorsal corticolimbic division provides the spatial, contextual organization that appears to be essential to implicit memory (Tucker DM, Luu P, unpublished observations).

This spatial, contextual memory system seems to operate with unique affective and cybernetic qualities. Studies of frontal lobe regulation of action have suggested that a unique form of control stems from the dorsal frontolimbic networks, involving a projectional, feedforward, and impulsive form of regulation both in human lesion studies (90) and in monkey lesion and electrical recording studies (91, 92). A converging perspective is provided by classical observations on emotional and personality changes with brain lesions in humans (93), which reveal that dorsal frontolimbic lesions lead to a *pseudodepression* syndrome, implying that a characteristic positive affect underlies the impulsive form of motor control in the dorsal frontolimbic division.

Early studies with intracranial electrode stimulation of the septal area of the Papez circuit in humans showed immediate increases in positive affect (as well as the propensity for sexual arousal) (94–96). More recent reports of intracranial stimulation during neurosurgery in humans have also observed enhanced positive affect from stimulation of the cingulate areas (97). The norepinephrine projections from the RAS have long been implicated in the positive affect of mania by the catecholamine hypothesis of the affective disorders (98). Arnsten and Goldman-Rakic (99) observed that projections from frontal lobe regulating the locus coeruleus (the brain stem source of norepinephrine projections) were from dorsal and medial regions of the frontal lobe and not orbital and ventrolateral frontal areas. Thus the dorsal division of the cortex appears to regulate, and be regulated by, the norepinephrine projections from the RAS, supporting not only the subjective experience of elation but also the inherent expansion of the scope of consciousness.

In a neuropsychological analysis of the implications of dorsal versus ventral limbic specialization for cognition and personality, Tucker and Luu (4) hypothesized that the motor, cognitive, and emotional evidence on the dorsal, archicortical limbic division is congruent with a feedforward, *impulsive* mode of motive control of cognition and behavior. When working memory is regulated by a habituation bias, the effect is an inherent selection for novel information, causing the mind’s current information to be expanded, even at the expense of explicit focal consciousness.

More recently, studying the continuity of the basal forebrain circuits supporting the dorsal limbic division, together with the dorsal distribution of norepinephrine and serotonin neuromodulators, Luu and Tucker have suggested how the vertical integration of the dorsal limbic control may draw on the RAS specifically (Luu P, Tucker DM, unpublished observations). Building from the evolutionary-developmental analysis of Butler (100), Luu and Tucker propose that the archicortical limbic division has a close relation to the reticular activating system of the lower brain stem through the lemnothalamic pathway that initially evolved to regulate the primitive general cortex (while bypassing the tectal midbrain, collothalamic, activation control centers) (101).

The three-layered primitive general cortex of reptiles is pyramidal, excitatory, and glutamatergic (102) and appears to have formed the basis for the pyramidal-dominant archicortical division (81, 83) of the six-layered mammalian neocortex (4, 103), contributing specifically to layers 1, 5, and 6. The specificity of brain stem norepinephrine projections for layers 1, 4, and 5, avoiding layers 2 and 3 specifically (104), may reflect the primordial lemnothalamic role of norepinephrine for the pyramidal-dominant archicortical architecture. The specificity of dorsal (and not ventral) frontal lobe projections to the locus coeruleus (99) is also compatible with the notion of a vertically integrated lemnothalamic control system that engages the Papez circuit and dorsal division of the neocortex (Luu P, Tucker DM, unpublished observations).

Although there are many questions that remain for a theoretical analysis, the specific form of neurophysiological regulation from a conserved lemnothalamic system, rooted in the neuromodulator projection systems from the reticular networks of the brain stem, may provide explanations for fundamental aspects of the affectively charged arousal control of waking consciousness (positive affect or elation) that is then integral to regulating the contextual memory of the archicortical division of the human cerebral hemisphere.

### Collothalamic Regulation of Tonic Activation, Negative Affect, and Ventral Limbic Control

The collothalamic, paleocortical limbic base of the ventral division of the mammalian cerebral hemisphere appears to be regulated by a different form of arousal control, *tonic activation* (4). Based in the midbrain dopamine projection system, the tonic activation system is not only integral to negative affect but has specific influences on the basal ganglia regulation of action and thought and on the tight focus of attention associated with the ventral limbic control of object memory.

The primary circuitry of the ventral limbic division has the amygdala at its center, with close links to the insula, basal ganglia, mediodorsal nucleus of the thalamus, and orbital frontal lobe. We hypothesize that the ventral limbic system is the telencephalic extension of the collothalamic system, which evolved with the tectal projections from the midbrain activation control center, mediated by dopamine (Luu P, Tucker DM, unpublished observations).

In the frontolimbic regulation of motor function, the ventral limbic division plays an important counterpart to the dorsal limbic control reviewed above. In controlling movements, the ventral limbic and orbital frontal networks provide *feedback* control, relying on the well-developed granular layer 4 of the ventral division to integrate sensory feedback guidance to align actions with the immediate contingencies of environmental contact (90). After the dorsal limbic influence has launched the action ballistically toward the goal (feedforward cybernetics of the impulse), the ventral limbic control provides the cybernetic *constraint* to align the action with sensory feedback, such as when the action approaches the goal and fine adjustments are required (105, 106).

In a similar vein, in the personality changes with frontolimbic lesions (93), orbital and ventral limbic lesions lead to *pseudopsychopathy*, a loss of normal social inhibition that may reflect the loss of constraint from ventral limbic control that is present in the intact brain. This constraint is theorized to be the regulatory influence of negative affect and anxiety, mediated by the claustro-amygdalar mechanisms of the ventral limbic division (4). This is a key mechanism of the focused attention of consciousness (107).

Thus the cybernetics of motive control may be integral to the unique form of object memory and cognition achieved in the ventral limbic division. The sustained focus of tonic activation operates to extract the object as the focus of reflection, through mechanisms of surround inhibition that isolate objects from their context in memory just as focal attention isolates objects in perception.

**Figure 3.**
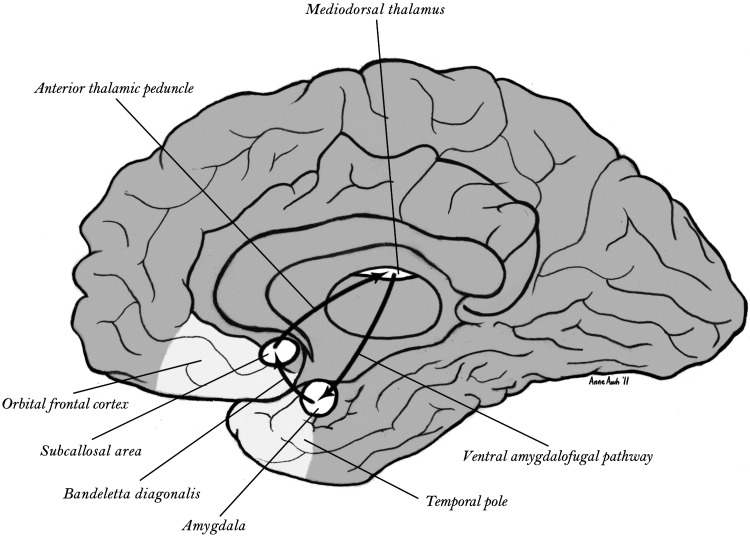
Triangular circuit integrating the paleocortical limbic division with 3-way reciprocal interconnections between the mediodorsal thalamus, anterior temporal lobe and amygdala, and orbital frontal lobe. From Tucker and Luu (4).

The sustained activity of the constraint function that generates object consciousness may be found in the *triangular circuit* (108) mediating reentrant connections between the ventral limbic and orbital frontal networks through the mediodorsal nucleus of the thalamus (Fig. 3). As Jones (108) observed, this triangular circuit is unusual in neurophysiology. The mediodorsal thalamus appears to control resonant, sustained activity integrating ventral limbic and orbital frontal network activity that may be integral to working memory for the objects of focal consciousness.

The influence of the dopaminergic midbrain tonic activation system in regulating this ventral limbic system, and the specific architecture of predictive coding, is suggested by the high density of dopamine projections to the orbital frontal lobe (104) and in layers 2–3 of the anterior cingulate cortex (104). The anterior cingulate cortex is the site of ventral limbic projections to the dorsal-archicortical cingulate cortex, such that feedback error monitoring in layers 2–3 would be particularly important to the limbic control of predictive coding across the corticolimbic hierarchy (5, 51).

The subcortical control of tonic activation appears to engage extensive cholinergic as well as dopaminergic regulation of the cortex (68). Although both cholinergic and dopaminergic projections are widespread, targeting dorsal as well as ventral cortical areas, the control of cholinergic projections from the forebrain nucleus basalis appears to be regulated by specifically ventral cortical areas of paleocortical derivation (109, 110). This specificity of control is consistent with the notion that the ventral limbic and striatal control over the cortex is grounded in a collothalamic system for regulating tonic activation (Luu P, Tucker DM, unpublished observations; Ref. 4).

### The Collothalamic Mechanism of Reentrant Basal Ganglia Control of Selection for Actions in Contexts

In humans, the basal ganglia provide multiple reentrant loops for regulating the development of actions, including the specification of a single action from an array of possible urges to action (59). Although often relegated to a noncognitive motor habit system, the mechanisms of basal ganglia motor articulation may be integral to more complex sequencing of cognition, including language (60). Remarkably, although the basal ganglia circuitry is specialized for reducing multiple urges into single actions, it also includes specific circuits for integrating contextual influences on action selection. A consideration of the neurophysiology of the basal ganglia, in close correspondence with the neocortical mechanisms of predictive coding, may suggest how the collothalamic mechanisms of the basal forebrain support the articulation and specification of focal objects of consciousness within the neocortical traffic.

In the organization of predictive coding across adjacent cortical networks (primary sensory/motor, unimodal association, heteromodal association, and limbic), the anatomy of the cortex provides for asymmetric reentrance, as mentioned above, in which the limbifugal (from limbic) projections arise from deep layers (5, 6) to project to the superficial layers (2–3) of the more eulaminate or external (closer to sensory/motor) network (50). In the applications of predictive coding theory to this architecture (51), the Bayesian predictions (expectancies) appear to be formed in deep infragranular output layers (5, 6) of the higher (closer to limbic) network and then project to the supragranular layers (2–3) of the lower (closer to primary sensory/motor) network (5).

Although predictive coding has been considered in relation to the neocortical architecture specifically, the evidence on control from the basal ganglia is relevant to specifying actions, and human neurodevelopmental evidence suggests it is critical to the specification of cognition as well (60). There are several basal ganglia organizational themes that provide key insights to the neurophysiology that allows incoming information to regulate action selection. To the extent that consciousness is organized from the inherent cognition for action or perception, these basal ganglia mechanisms may explain the (largely unconscious) articulation of explicit objects of conscious experience.

The differential influence of lemnothalamic and collothalamic regulation can be analyzed in the different evolutionary origins for different cortical layers, with layers 2–3 being derived from the collothalamic trend, whereas layers 1, 5, and 6 are derived from the lemnothalamic trend (the original 3-layered cortex) (111). Within the canonical cortical microcircuit (CCM) columnar organization of cortical layers, the upper and lower aspects of layer 5 (5a and 5b, respectively) represent two rather distinct microcircuits that may explain how contexts represented in the neocortex may influence the reentrant basal ganglia circuits that progressively select specific actions from an array of possibilities.

Upper layer 5a neurons connect to layers 2–3 and deep layer 5b neurons. Deep layer 5b cells also receive inputs from layers 2–3 and 5 neurons, but they do not provide inputs to the 2–3/5a CCM. Layer 5b pyramidal cells project outside of the telencephalon (e.g., to the spinal cord) and are referred to as *pyramidal tract* cells. In contrast, pyramidal cells of layers 2–3 and 5a are *intratelencephalic*.

From a predictive coding perspective, layer 5b, because of its connection with layer 2/3, may be related to direct movement planning, particularly in response to current sensory inputs. Both Bayesian state/causes of sensory inputs (layer 3) as well as the predictions (explanatory models) of those sensory inputs (layer 5a) represent affordances (or more broadly action plans when considered across a network of brain structures) of sensory representations (112). Importantly, this state/prediction CCM represents information that does not leave the telencephalon. Complementing the state/prediction CCM, 5b cells receive processed inputs from layer 2–3 neurons as well as the prediction of these states by 5a cells, and their outputs reflect motor efference, or, in other words, predictions (113).

The striatal regulation of specific CCMs differs between the patch and matrix compartments of the striatum. These compartments are considered to be somewhat independent channels (114–116). The patch (i.e., striosome) compartment receives cortical inputs from layer 5b projections and in turn projects to the substantia nigra compacta (SNc) (114, 117), which supplies dopamine (DA) input into the striatum. The matrix compartment receives primarily intratelencephalic inputs (3/5a), particularly from layer 5a in primates (116), but the matrix also receives some 5b inputs.

The output of the striatum is divided between the classic direct and indirect pathways of cortical-basal ganglia circuitry. Here the evidence shows that intratelencephalic neurons preferentially target the direct pathway, whereas the pyramidal tract neurons preferentially target the indirect pathway (116).

A general theory of basal ganglion function must consider the extensive inputs to the basal ganglia that are then funneled into limited outputs. This funneling of information presents a challenge for encoding of information and requires some form of dimensionality reduction to effectively encode relevant information. Different models propose different mechanisms for data reduction, for example selection of motor plans (118).

Of particular relevance to the focusing of attention is Shipp’s recent model that describes how contextual inputs are considered within the basal ganglia for action selection (150). In this model, action plans (i.e., a bid for action) arrive in the striatum through the classic closed-loop circuitry (from cortex to striatum to basal ganglia output nuclei to thalamus and back to the originating cortical site). Context is provided by the circuitry of the basal ganglia patch regions, the substantia nigra pars compacta, connected to the striatum and cortical regions that that form closed-loop inputs. As action plans arrive in the striatum (matrix compartment), they project to both the direct and indirect pathways. Through the cortical microcircuits and projections described above, context inputs are available to both direct and indirect pathways. It is thought that the direct and indirect pathways capture the positive and negative values of an action plan from prior reinforcement history, as regulated by dopamine activity.

The tonic activation cybernetics of dopaminergic activity (39) produces a redundancy in motor selection, such that those action plans that have been associated with reward will likely occur again and those that are not will be inhibited (118). The result of this sustained focus, elaborated through extended cortical-thalamic-basal ganglia-midbrain loops, is the ability to draw on memory of reinforcement, through progressive reentrant cycles, to select one action plan from many. The balance of positive and negative values associated with an action plan is thus learned implicitly, and this history of experience forms the contextual basis for the more explicit selection of a focused action plan.

Relative to the process of focusing consciousness from a broad field, it is remarkable that context/predictions, as conveyed by either 5a (intratelencephalic) or 5b (pyramidal tract), are of lemnothalamic origin, and yet they are action inputs to the basal ganglia (a key collothalamic telencephalic target). A key theme in the evolution and development of the mammalian neocortex is this weaving of lemno and collo regulation in the six-layer architecture.

It appears to be through the convergence of the lemnothalamic predictive/contextual inputs (forming the field of consciousness) that the collothalamic dopaminergic activity then enables selection (via balancing the direct and indirect pathways) of the final action that comes to be experienced as the focused action sequence. By integrating the patch and matrix basal ganglia controls with the specific predictive coding circuitry of the neocortex, it thus may be possible to consider how selection for action is achieved by ventral limbic dopaminergic activity regulating basal ganglia control. These neurophysiological mechanisms may describe not only the selection of actions through habits and reinforcement but the differentiation and focus of object memory, parsing significant objects from experience. This conservation of elementary neurophysiological mechanisms for action selection may underlie the progressive specification of focal consciousness.

### Dual Limbic Modes for Reentrant Monitoring of Awareness?

Thus there are unique subcortical control systems, anchored in the collothalamic and lemnothalamic control circuitry of the midbrain and brain stem activation and arousal control systems, that appear to regulate the unique reentrant networks of the dorsal limbic Papez circuitry (Fig. 2) and the ventral limbic triangular circuit (Fig. 3) that form the major memory systems of the mammalian, and human, brain. The basal ganglia reentrance to the cortex involves important additional channels that can be thought of as extending the ventral limbic constraints to focus the span of information that is articulated into specific thoughts as well as specific actions. Understanding the unique cybernetics of feedforward (dorsal) and feedback (ventral) control in these networks may provide greater specificity to the notion that the limbic control of the neocortical hierarchy of networks (with unique reentrant, cross-laminar patterns of network connectivity) could support properties of conscious monitoring of cognition (5, 17).

Importantly, whether conscious or not, the reentrant property of maintaining activity in these dual limbic circuits has differing implications not only for the scope of working memory but also for the affective quality that is essential to the subjective meaning of experience. The elation associated with strong phasic arousal, whether normal (46) or abnormal (77), expands the scope of working memory, and thus conscious experience. This leads to enhanced flexibility and creativity, but also, in the extreme cases of elation in mania, to the *flight of ideas* (76). In contrast, the anxiety associated with strong tonic activation leads to a tight focus of consciousness (119, 120), which in pathological extremes leads to the rigidity and stereotypy of thought and action in obsessive-compulsive and paranoid disorders (39, 70, 76).

There are thus well-known altered states of consciousness that are created by abnormal elation or pathological anxiety. However, the limited introspection inherent to human consciousness is evidenced by the fact that these states are not associated with subjective insight. In a manic state, the person makes major life decisions with unfounded optimism, with little insight into how the present mood structures the appraisal of uncertainty (76, 121). Similarly, the anxious person is preoccupied by obsessive or paranoid thoughts, with little insight into the qualitative motivation (and uncertainty enhancement) that is applied by the present mood to the process of consciousness (119, 120).

The foundation of these affective-cognitive states of consciousness in the brain stem and midbrain mechanisms of arousal and activation is perhaps most clearly evidenced by examples of stimulant drug abuse, in which the motivation to achieve a state of elation leads to ingestion of drugs such as cocaine and amphetamine that have an immediate effect of both norepinephrine and dopamine release (122, 123). Apparently because of its inherent habituation bias, the elation from stimulant abuse quickly fades, whereas, because of its redundancy or sensitization bias, the anxiety remains. Of course, as the noradrenergic elation is exhausted, the PA dimension reduces to depression (the crash of the drug high). At the same time, the continued stimulant drug use leads to increasingly less elation and more anxiety. The result is the *strung-out* state, characterized by obsessive actions, paranoid ideation, and an obsession with seeking the next drug ingestion in a cycle of addiction (39, 124).

Thus, the structure of consciousness is regulated inherently and predictably by the elementary neurophysiological control systems of tonic activation and phasic arousal. The theoretical insight that these differentially regulate ventral limbic object memory and dorsal limbic contextual memory may suggest how specific memory systems support differing biases in the structure of awareness. Yet there are many examples in which human self-regulation of these control systems involves little or no conscious insight.

This automatic or implicit regulation of cognition by adaptive limbic concerns may be evidenced most clearly by what appears to be the differential consolidation of lemnothalamic and collothalamic contributions to memory in the unique neurophysiological mechanisms engaged in the stages of human sleep. Although the unique neurophysiology of sleep stages is largely unconscious, we propose in consolidating implicit and explicit forms of memory in sleep that sleep may play a central role in the process of developing explicit focus of object consciousness, as well as in the more implicit functions of memory and cognition in supporting the broader contextual field in which consciousness arises (12).

## CONSOLIDATING IMPLICIT AND EXPLICIT FORMS OF MEMORY IN SLEEP

The concept of lemnothalamic and collothalamic regulatory systems builds on the recognition that certain modes of regulating neural activity arose early in vertebrate evolution in the RAS of the lower brain stem (forming the base of the lemnothalamic system) and, apparently later in evolution, the collicular regions of the midbrain (the base of collothalamic regulation). These differing forms of arousal control provided fundamental neurocybernetic properties that were retained at each succeeding stage of neural evolution. Remarkably, these regulatory modes were apparently incompatible to some extent, because they were incorporated in relatively separate diencephalic and telencephalic connections and functions in mammalian evolution: the lemnothalamic and collothalamic systems. A key neurophysiological function in which these regulatory systems operate relatively independently is the stages of memory consolidation in sleep.

We theorize that the lemnothalamic regulatory system, mediated by norepinephrine, serotonin, and cholinergic neuromodulators, underlies the control of the dorsal limbic system and associated dorsal (archicortical) neocortex (Luu P, Tucker DM, unpublished observations; Ref. 5). Importantly, this control system and its archicortical targets may be responsible for the unique neurophysiology of rapid eye movement (REM) sleep and the implicit memory that is consolidated in this stage of sleep.

In contrast, we propose that the collothalamic regulatory system, mediated primarily by the dopamine projection centers in the midbrain, underlies the tonic activation integral to the ventral limbic system, centered on the claustro-amygdalar complex, with integral input to the basal ganglia, as well as general support for the ventral (paleocortical) division of the neocortex (Luu P, Tucker DM, unpublished observations; Ref. 5). Several lines of evidence suggest that the collothalamic system is exercised uniquely in non-REM (NREM) sleep to consolidate the explicit memory of the human brain that is particularly important to the more explicit, definite, and communicable contents of conscious experience.

### The Lemnothalamic Control of REM Sleep

As noted above, an important finding for understanding the specificity of neuromodulator action within the RAS was the discovery that the onset of REM sleep is associated with a suppression of norepinephrine and serotonin activity and an enhancement of cholinergic regulation of the forebrain (1, 125). An alternative view has been provided by Solms (126), who emphasizes the possible role of dopamine in REM regulation.

Consistent with the RAS regulating the lemnothalamic system, the PGO waves that are characteristic of REM sleep (127) are restricted to the lemnothalamic (pontine, geniculate, occipital) circuitry.

Particularly in rodents, REM sleep is associated with theta rhythms throughout cortical and limbic networks. Analysis of the subcortical generators of these rhythms shows them to be restricted to lemnothalamic and not collothalamic regions. Within the brain stem reticular formation, the nucleus pontis oralis is responsible for the static (nonrhythmic) firing underlying the theta activity in the forebrain. The conversion of this tonic firing to rhythmic discharges at the theta frequency is due to activity of the supramammillary nucleus of the hypothalamus, which then resonates with septal, hippocampal, and other structures of the Papez circuit in maintaining the limbic theta oscillation (125, 128).

A demonstration of the lemnothalamic, archicortical specificity of REM sleep has recently been achieved by Luppi and associates with optogenetic methods in rodents. They observed that REM sleep was associated with multiple components of the Papez circuit, including the anterior cingulate cortex, retrosplenial cortex, medial entorhinal cortex, and dentate gyrus of the hippocampus (129). The importance of this archicortical circuitry to spatial, contextual memory is well known, and disruption of the hippocampal theta rhythm with optogenetic block of GABA regulation from the medial septum has been shown to create the predictable behavioral deficit in spatial memory (130).

A link between the archicortical lemnothalamic circuitry and the functional integration of hippocampal with cortical activity in REM was recently suggested by Del Rio-Bermudez and associates (131), who observed that whisker stimulation during REM in 7- to 9-day-old rat pups resulted in synchrony between the hippocampus and primary somatosensory cortex. This synchrony was disrupted when reafference was blocked with transection of the infraorbital nerve, implying that the activity-dependent exercise of hippocampal-cortical connectivity in early development is engaged specifically in REM sleep.

In humans, a number of limbic structures have been observed to be active in REM sleep in cerebral blood flow studies (132). Localization of the sawtooth waves of REM sleep with high-density EEG has recently suggested that these features of REM neurophysiology are localized to the cingulate cortex (133), consistent with the proposal that the archicortical dorsal limbic circuit is specifically consolidated in REM sleep.

### The Neurodevelopmental Consolidation of Implicit Memory and the Field of Consciousness in REM Sleep

For many years, it has been clear that sleep must have a function in neural development and that this function is operative in the beginning of brain development, in utero. The fetus shows active and quiet stages of brain activity that appear to reflect the developmental origins of the active (REM) and quiet (NREM) stages of mature sleep after birth (134). When Jouvet (135) observed the origins of REM sleep in the fetus, he proposed that this must reflect early exercise of instinctual patterns in brain development.

As recent research has clarified the role of REM sleep and its vivid dreams in memory consolidation, it has become clear that REM sleep contributes little to the explicit recall that we may associate with declarative or explicit memory, which instead is consolidated in NREM sleep (136). Rather, the neurophysiology of REM is important to both emotional integration (57, 137) and the consolidation of implicit memory (136).

Examples of implicit memory include solving insight problems (54, 56), in which the solution is not consciously apparent but appears to be organized intuitively, in the unconscious background of cognition, before finally becoming explicit to conscious awareness. For understanding the neurophysiological mechanisms of consciousness, the implications of this research may be important. What appears to be the fundamental role of REM sleep and dreams in organizing neural development and ongoing cognition turns out to be important not to the explicit recall of information in focal consciousness but to the more general integration of meaning in the implicit field of the mind from which intuition and insight must arise.

### The Collothalamic Control of NREM Sleep

The primary neurophysiological features of NREM sleep that appear important to memory consolidation are the spindles that dominate in stage N2 sleep and the slow oscillations that become strong and synchronous in stage N3 sleep (54). The collothalamic projections to ventral limbic regions, including the foundational control of the telencephalon by the claustro-amygdalar complex, may best be understood through understanding the evolutionary context. In reptiles the collicular projections to the anterior dorsal ventricular ridge (ADVR), described by Nauta and Karten (103) as the *external striatum*, are involved in regulating slow oscillations and spindles attributable to this structure (138, 139). With the apparent incorporation of the ADVR in the six-layered neocortex in mammalian evolution (4, 103, 140), the collothalamic control was extended to the subcortical claustro-amygdalar complex and the ventral limbic-cortical division that it regulates (Luu P, Tucker DM, unpublished observations).

Consistent with this evolutionary anatomical analysis of collothalamic origins, and the proposal that collothalamic control is the foundation of NREM sleep, recent optogenetic studies in mice have found that the slow oscillations of NREM sleep are regulated by discharges in the claustrum (141). Furthermore, high-density EEG studies of human slow oscillations in NREM sleep in our laboratory have recently shown that these large discharges of deep NREM sleep are typically localized to ventral limbic (medial anterior temporal and hippocampal gyrus) and caudal orbital frontal regions (142, 143). These are integral forebrain structures of the collothalamic system, with their regulatory foundations in the midbrain structures of the collicular-ventral tegmental area.

### The Neurodevelopmental Consolidation of Explicit Memory and the Focus of Consciousness in NREM Sleep

As noted above, the striking realization from recent studies of memory and sleep is that the capacity for explicit memory, the previous experience that can be recalled consciously, requires the consolidation provided by NREM stages of sleep (54, 136). If it is correct that REM sleep reflects dominant consolidation activity in the lemnothalamic, dorsal limbic division whereas NREM sleep reflects the specific consolidation within the collothalamic, ventral limbic division, this realization may have implications not just for the neurophysiology of sleep but for the neurophysiology of waking consciousness that must integrate both the implicit field and the explicit focus of consciousness.

A recent interpretation of sleep consolidation findings in children by Born, in a commentary on Wang et al. (144), provides a way of understanding the maturation of cognition in relation to both dorsal and ventral memory circuits that may explain developmental changes in consciousness as well as sleep. The findings showed that sleep consolidation in children supported both explicit (recall of specific word pairs) and implicit (recall of which list the pairs happened to be in) but not improved recall of the word pairs independent of the implicit context (list). Adults, in contrast, showed sleep-enhanced recall of the specific word pairs regardless of whether they recalled the list they happened to be in (144).

Born’s interpretation considers the theoretical differentiation of memory between dorsal representation of context and ventral representation of specific objects in relation to hippocampal circuits as hypothesized by in a series of papers by Eichenbaum and associates (145, 146). Consistent with the ventral limbic contribution to object memory, a hippocampal circuit through the perirhinal cortex is important to mediating specific item (object) memory. Consistent with the dorsal limbic regulation of context memory, a separate hippocampal circuit through parahippocampal cortex supports spatial (where or when) memory capacity. Importantly, Preston and Eichenbaum (146) identified medial prefrontal circuits that mediate between the context and object circuits to achieve executive control for the delineation of objects in their embedding contexts.

Born’s interpretation of the children’s sleep and memory performance data points out that, without the adult’s maturation of frontal regulatory capacity, the child’s memory function is more holistic, with items not so clearly delineated from the contexts in which they were experienced (147). The effect is reminiscent of Heinz Werner’s (148) emphasis on the *syncretic* nature of the child’s experience, in which poorly differentiated implicit associations are fused in a holophrastic amalgam. The interesting implication is that the increasing executive self-regulation of the adult creates a more differentiated quality of cognition, with increased capacity to separate, and remember explicitly, the discrete objects of experience.

Particularly relevant to the contextual basis for consciousness, Born points to Eichenbaum’s (149) reasoning that the dorsal, archicortical (hippocampal) contextual memory system is as important for the temporal context for behavior as it is for the spatial context. Applied to William James’s analysis of consciousness (13), the ongoing lemnothalamic memory for the temporal context may support the implicit meaning that organizes a more extended apprehension of the ongoing stream of consciousness. In contrast, when the increasing specification of object consciousness is achieved by the ventral division and its associated striatal controls, the effect in time is not only to sustain the object focus but to suppress awareness of the more temporally extended stream of consciousness.

## TOWARD A NEUROPHYSIOLOGICAL PHENOMENOLOGY

There thus may be interesting implications of the rapidly developing insights into sleep for understanding not only the neurophysiology of memory consolidation but also the neurophysiology of the process of consciousness. We have reviewed evidence that the dual dorsal and ventral limbic memory systems, drawing on the respective vertical integration of lemnothalamic phasic arousal and collothalamic tonic activation control systems, provide unique ways of organizing experience to respond to adaptive motives, both in the background of unconscious processing and in the foreground of what appears in awareness. Until we study them, we may have little conscious awareness of these adaptive neurophysiological mechanisms, except for the intrinsic feelings they engender. Yet perhaps by studying them we could aspire to a more extended, neurophysiological, phenomenology.

The focus of consciousness (12) appears from the current evidence to be achieved largely through the collothalamic mechanisms of object memory, as ventral limbic networks narrow the scope of attention through reentrant engagement of redundancy and selection, achieving the surround inhibition in semantic as well as sensory-motor space by the action selection capacities of the basal ganglia.

The field of consciousness (12), on the other hand, appears to be maintained as a dynamic and holistic context of information access through the lemnothalamic mechanisms of context memory. Elaborated in the dorsal corticolimbic division, this context memory appears to be integral to the cognitive span of time as well (149), extending the continuity of experience in the ongoing stream of consciousness. The uniquely implicit memory of the dorsal limbic division is consolidated each night through the specific neurophysiology of REM sleep, organizing generative and imaginative capacities that appear essential to develop the intuitive contents of consciousness. The brain’s lemnothalamic capacities, enacted by the inherent habituation cybernetics of phasic arousal, are thus fundamental to the process of consciousness, but they seem to remain in a generative, intuitive role. They in fact recede from awareness when the focus of consciousness is sharpened by the differentiation and articulation of the feedback cybernetics of collothalamic tonic activation.

### Neurophysiological Mechanisms of the Ineffable Quale

Considering the broader neurophysiological mechanisms of memory and cognition, we suggest that Chalmers has defined the hard problem of consciousness through an abstract characterization of experience that isolates the subjective percept (and its abstracted qualia) from the ongoing phenomenal flow of consciousness. The fact that so many philosophers and neuroscientists have resonated to his account of experience implies that the ineffable quale is easily summoned in the minds of disciplined thinkers. So, for example, we may reflect on the subjective feeling of a middle C, extracting it from ongoing memory or perception as an abstract construction. Chalmers argues that no matter how much neurophysiological evidence you might have about the neural mechanisms giving rise to that particular quale, you can never explain why that tone feels the way it feels.

We propose that the specific process of consciousness that generates the quale might be described as *pristine subjectivity*, a highly focused extraction of a discrete perceptual quality from the more embodied stream of consciousness. Furthermore, imagining the “something that it is like” to have this experience invokes the self having the experience, the personal perspective of experiencing the quale. The simple perceptual event is thereby endowed with rich, implicit, personal meaning.

And yet, because the focus on this object of consciousness has become intense, the process of cognition itself, particularly the implicit associations that imbue the quale with subjective significance, are suppressed from consciousness. Your perception of middle C may be colored by the pianos of your childhood or the specific music you have learned that was organized around the harmonics with this tone. These implicit meanings at the fringe of your conscious experience remain integral to the significance that you feel with the percept, until a highly focused object consciousness applies its surround inhibition to suppress the implicit associations from present awareness.

Considering the neurophysiology of consciousness to include the implicit contextual memory of the dorsal division as well as the explicit object constructions of the ventral division, we can reembed the experience of middle C in the full phenomenology of conscious meaning—field, fringe, and focus—in which subjective meaning must include multisensory, motor, and affective dimensions of personal experience, each of which can be informed by neurophysiology. Then we have all we need, or can have, for an explanation of why this experience has the meaning it does and feels as it does. The hard problem can exist only under conditions that mistakenly isolate qualitative experiences as abstracted figments, semantic islands frozen in the stream of embodied consciousness.

The study of neurophysiology is thus not only relevant to understanding subjective experience more broadly but might be able to explain how the isolated qualia of consciousness could be constructed and believed. The collothalamic mechanisms for delineating objects of perception and memory in the ventral corticolimbic division operate to separate a discrete object from its contextual surround, in time and space. The discreteness may operate in interoception, what we feel, as well as exteroception, what we hear. The inhibition of surrounding associations in focal object perception is achieved through the redundancy bias of tonic activation, and it may be sustained by the reentrant specification through subsequent basal ganglia loops, just as in action selection. In the quale of middle C, the feelings and multiple personal meanings may continue to give significance to this instance of consciousness. Yet the porous, malleable boundary between focus and fringe results in the nonconscious character of fringe meaning, and this creates the illusion of an independent quale, or felt state, that is unconnected from underlying neurophysiological mechanisms. Once suppressed by an overly focal object consciousness, the contextual influences of implicit memory may become fully unconscious, apparent in introspection only as they give a magical feeling to the discrete subjective percept. The ineffable quale then emerges when the ongoing stream of consciousness becomes so focused and sustained as to create the subjective illusion of singularity.

## DISCLOSURES

No conflicts of interest, financial or otherwise, are declared by the authors.

## AUTHOR CONTRIBUTIONS

D.M.T., P.L., and M.J. conceived and designed research; D.M.T., P.L., and M.J. edited and revised manuscript; D.M.T., P.L., and M.J. approved final version of manuscript.
